# Elevated NT‐proBNP in the Setting of Ongoing Chest Infection Leading to Incidental Diagnosis of Infective Endocarditis—A Case Report

**DOI:** 10.1155/cric/8004408

**Published:** 2026-09-28

**Authors:** Jaiden Townsend, Tiago Correia da Silva, Salman Nishtar, Ibrahim Antoun

**Affiliations:** ^1^ Division of Cardiovascular Sciences, University of Leicester, Leicester, UK, le.ac.uk

**Keywords:** blood culture–negative endocarditis, cardiac implantable electronic device, infective endocarditis, lead vegetation, NT-proBNP, pacemaker, transoesophageal echocardiography, transvenous lead extraction

## Abstract

Infective endocarditis (IE) poses a known diagnostic challenge due to its variable clinical presentation. High suspicion for IE is generally driven by fever and positive blood cultures in the absence of an alternative focus of infection. We report an adult woman with a permanent pacemaker who presented with persistent fever, pleuritic chest pain and productive cough, diagnosed with right‐sided community‐acquired pneumonia. Blood cultures were negative; blood tests showed elevated inflammatory markers and an elevated white blood cell count, consistent with ongoing infection; and the NT‐proBNP (obtained due to shortness of breath and evidence of pleural effusion on chest X‐ray [CXR]) was markedly elevated, prompting transthoracic echocardiography (TTE). TTE demonstrated a large, irregular, independently mobile mass adherent to the pacemaker lead within the right atrium, prolapsing across the tricuspid valve into the right ventricle during diastole (≈2.3 × 1.0 cm), mild tricuspid regurgitation and normal biventricular systolic function. Transoesophageal echocardiography (TOE) confirmed a similar lead‐adherent structure (≈2.5 × 1.0 cm), supporting a diagnosis of pacemaker‐related IE in the appropriate clinical context. The patient was referred for complete device extraction at a specialist centre. This case highlights the diagnostic and management challenges of blood culture–negative endocarditis, the central role of echocardiography in the diagnosis of IE and the importance of integrating and interpreting imaging findings in the wider clinical context.

## 1. Introduction

Infective endocarditis (IE) is a significant public health challenge characterised by high morbidity and mortality. It remains a diagnostic challenge because of its highly variable clinical presentation, which can mimic a range of other medical conditions, including rheumatological, neurological and common infectious disorders. Diagnosis is traditionally driven by high clinical suspicion, often triggered by fever and positive blood cultures in patients with known risk factors [1].

In this context, blood culture–negative endocarditis (BCNE) presents a particular management hurdle. BCNE is increasingly approached as a syndromic diagnosis requiring systematic evaluation rather than as a single entity [2, 3]. Blood cultures may remain negative because antibiotics were given before cultures were collected and/or because causative organisms are fastidious or require specialised testing, complicating pathogen‐directed therapy and increasing reliance on broad empiric regimens with attendant adverse effects [1, 2].

Cardiac implantable electronic device (CIED) infections span a clinical spectrum from localised pocket infection to systemic infection involving intracardiac leads and/or valves [1, 4]. CIED‐related IE is clinically high‐stakes because durable cure typically requires complete system removal in addition to antimicrobial therapy, and delays in recognition may worsen outcomes [1, 4]. Contemporary recommendations emphasise multidisciplinary care pathways (often operationalised as ‘endocarditis team’ models), early imaging with echocardiography and timely extraction when systemic CIED infection is confirmed [1, 4].

The present case highlights the diagnostic and management challenges posed by suspected CIED‐related IE in the context of persistently negative blood cultures and competing clinical diagnoses. We describe an adult patient presenting with systemic symptoms initially attributed to respiratory infection, in whom transthoracic echocardiography (TTE) and transoesophageal echocardiography (TOE) demonstrated a large, mobile pacemaker lead–associated right atrial mass. This report emphasises the central role of echocardiography in detecting clinically significant lead vegetations, the importance of integrating imaging findings with systemic inflammatory features in differentiating infectious from noninfectious lead‐associated masses and the practical implications for antimicrobial stewardship and device extraction planning.

## 2. Case Presentation

An adult woman with a permanent pacemaker presented with fever, pleuritic chest pain and a productive cough with yellow sputum. Past medical history included chronic obstructive pulmonary disease, Type 2 diabetes mellitus, hypertension and significant psychiatric comorbidity. Social vulnerability and safeguarding needs were identified during admission; all such details are anonymised in this report.

During hospitalisation, she was treated for right‐sided community‐acquired pneumonia with clinical improvement. She developed *Clostridioides difficile*–positive diarrhoea that resolved with therapy. Despite improvement in respiratory symptoms, she remained systemically unwell with ongoing pyrexia and persistently elevated inflammatory markers. In parallel, due to initial presentation of shortness of breath and evidence of a unilateral effusion on chest X‐ray in the context of a patient with cardiac history and risk factors, NT‐proBNP was also requested and was found to be significantly elevated. This prompted a referral for an inpatient TTE.

Multiple blood cultures were obtained during the illness course due to persistently elevated inflammatory markers and pyrexia, but they remained negative despite antecedent and ongoing antimicrobial exposure. As prior (antecedent) antimicrobial therapy is the commonest cause of culture‐negative endocarditis, it is the most likely explanation for the persistently negative cultures in this patient. There was no reported recent vascular access, dental or skin procedure which could represent an alternative bacteraemia source.

TTE demonstrated a large pacemaker lead–associated intracardiac mass, and referral was made for transfer to a specialist centre for complete device and lead extraction, consistent with standard approaches to systemic CIED infection [1, 4].

## 3. Echocardiographic and Imaging Findings

### 3.1. TTE

In late December 2025, TTE demonstrated nondilated right‐sided chambers with preserved right ventricular systolic function. A pacing lead was visualised within the right atrium and right ventricle. A large intracardiac structure with irregular contours and independent mobility appeared adherent to the pacemaker lead inside the right atrium, located immediately inferior to the septal leaflet of the tricuspid valve and prolapsing through the tricuspid annulus into the right ventricle in diastole (Figure 1). The maximal measured dimensions were approximately 2.3 × 1.0 cm (Figure 2).

**Figure 1:**
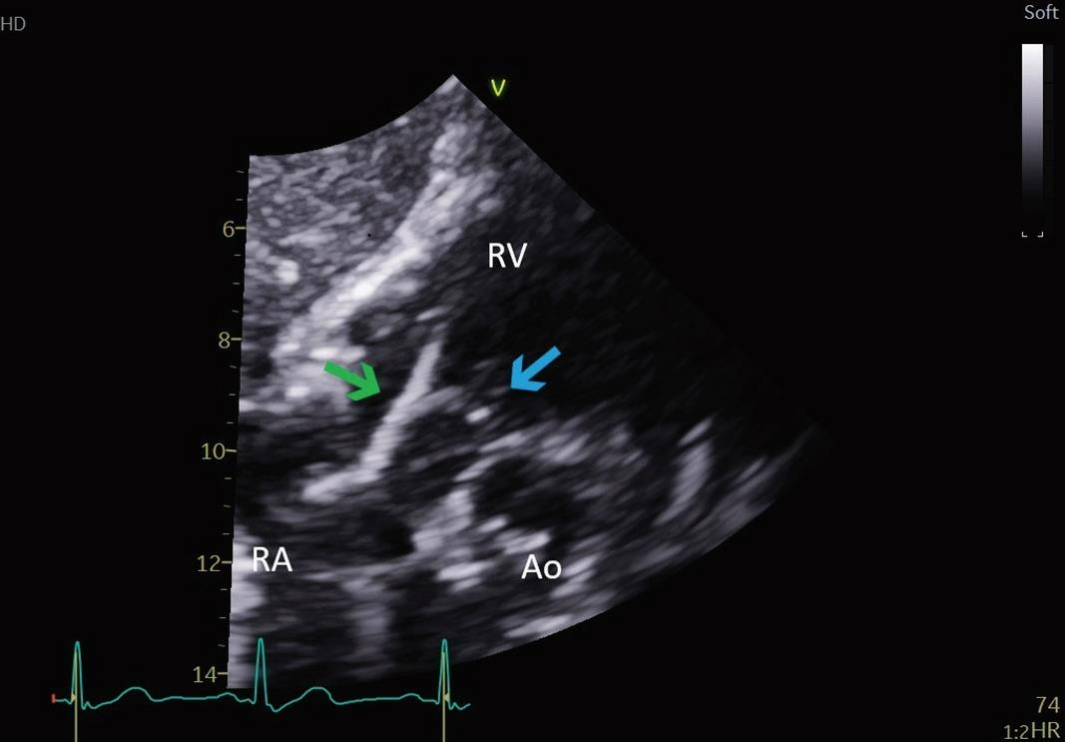
TTE image: subcostal view, right heart focused, demonstrating the pacemaker lead (green arrow) and an independently mobile, rounded structure adherent to the lead (blue arrow). RA, right atrium; Ao, aortic root; RV, right ventricle.

**Figure 2:**
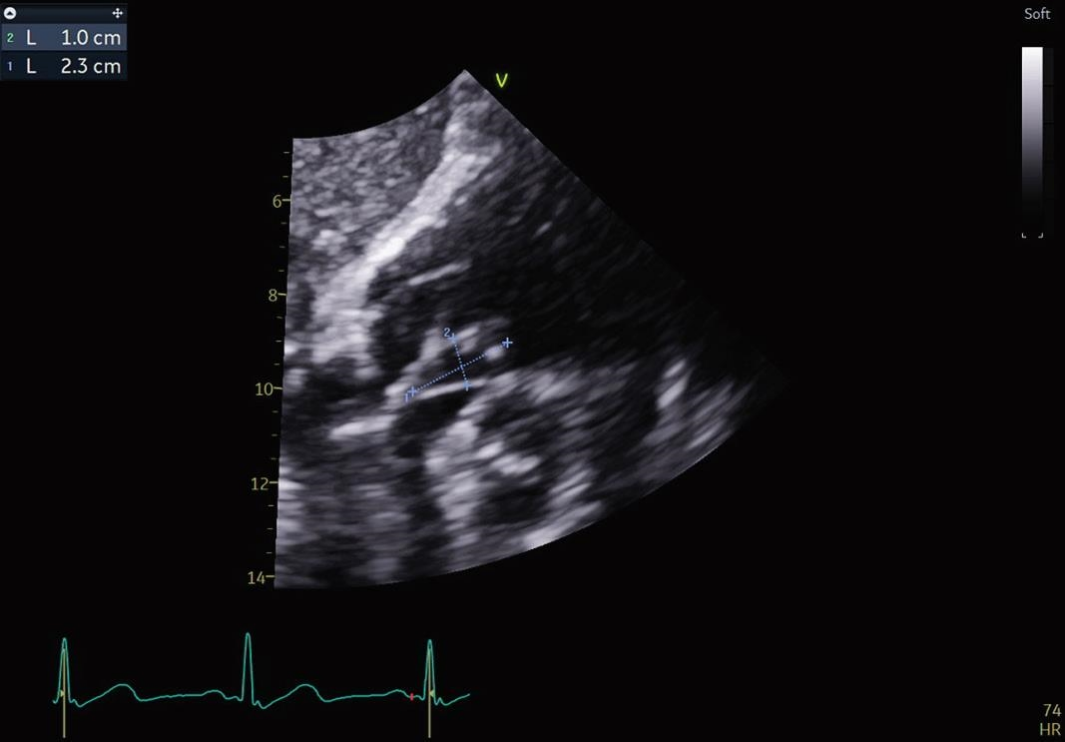
Same view as depicted in Figure 1, measuring the dimensions of the reported lead‐adherent mass.

Left ventricular size and systolic function were normal, with a biplane ejection fraction of 58%, with impaired diastolic function and elevated left ventricular filling pressures. Mild aortic stenosis with mild eccentric aortic regurgitation was reported, with minimal mitral regurgitation. Mild tricuspid regurgitation was present, with estimated pulmonary artery systolic pressure approximately 32 mmHg plus right atrial pressure (< 5 mmHg).

Importantly, the abnormal TTE findings prompted immediate reconsideration of the working diagnosis and led directly to escalation of antimicrobial therapy for suspected IE.

### 3.2. TOE

Following the abnormal TTE findings, TOE was performed to provide a more detailed assessment of the intracardiac mass and evaluate the extent of cardiac involvement.

A large, intracardiac lead‐adherent mass can be seen in the right atrium, directly superior to the septal leaflet of the tricuspid valve. The structure exhibited irregular contours, independent mobility and a heterogeneous appearance (Figures 3 and 4). The measured size was approximately 2.5 × 1.0 cm. Given the anatomical appearance, location and broader clinical context, this structure was interpreted as being consistent with vegetation due to IE. TOE also reported mild‐to‐moderate eccentric tricuspid regurgitation with higher estimated pulmonary artery systolic pressure (~50 mmHg plus right atrial pressure).

**Figure 3:**
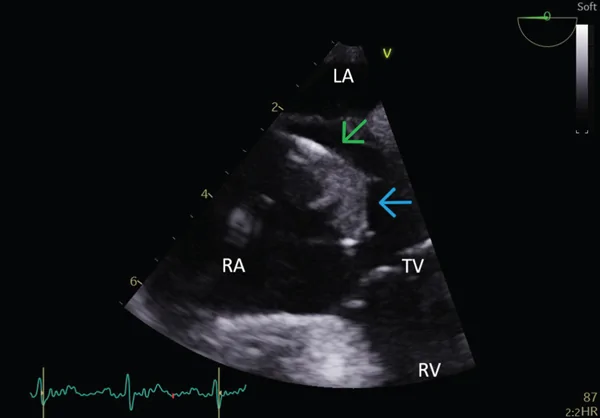
TOE image: 4C view, focused right atrial view, displaying the pacemaker lead (green arrow) and an irregularly shaped, independently mobile, echodense structure adherent to the pacing lead (blue arrow). LA, left atrium; RA, right atrium; TV, tricuspid valve; RV, right ventricle.

**Figure 4:**
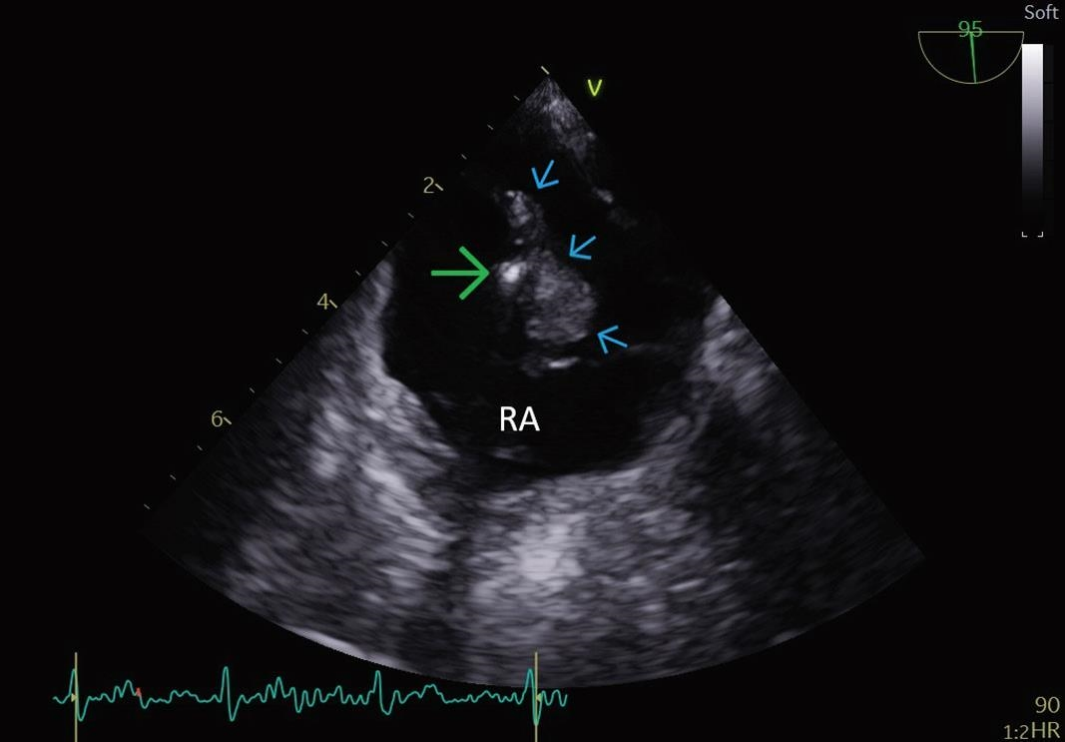
TOE image: focused right atrium view (95°) depicting the pacemaker lead (green arrow, coronal view) and the reported irregular‐shaped, independently mobile, echodense structure adherent to the pacing lead (blue arrows). RA, right atrium.

## 4. Discussion

The present case illustrates how IE can present with a variety of nonspecific symptoms and that particular attention is warranted for patients with ongoing pyrexia despite antibiotic use, particularly in the context of CIED [1].

In this reported case, initial TTE demonstrated a large lead‐adherent mass after careful examination. However, echocardiographic interpretation requires caution: Lead‐associated echodensities can be incidental and noninfectious, and morphology alone may not reliably distinguish infectious vegetations from thrombus/fibrin or encapsulation [5, 6]. Contemporary observational data show that incidental lead‐based masses are common on TOE performed for noninfectious indications and that minimal morphologic differences may be present between infectious and noninfectious lead echodensities [5, 6]. Accordingly, imaging must be integrated with systemic features (e.g. persistent fever and elevated inflammatory markers), microbiology and the overall clinical context [2, 4]. Interpretation in this case was particularly challenging, as the TTE indication was initially just an elevated BNP; however, after reviewing the patient′s notes, it was evident that there was an ongoing infection, which led the TTE report to suggest IE as the cause of the presentation. It should also be made explicit that tricuspid regurgitation severity was not the primary driver of clinical concern in this case and remained mild to mild–moderate on serial echocardiography. Rather, the trigger for cardiac imaging was the markedly elevated NT‐proBNP in the context of dyspnoea and pleural effusion, independent of the degree of valvular regurgitation.

Applying the 2023 Duke–ISCVID criteria, this case met one major criterion (echocardiographic evidence of a vegetation on the intracardiac lead) and two minor criteria (a predisposing CIED and fever ≥ 38°C), satisfying the threshold for possible IE. As all blood cultures were negative, no microbiological criterion was met, and a definite classification would have required microbiological or histopathological confirmation from the explanted lead. Importantly, persistent fever in a patient with a CIED already represented two minor criteria and, in itself, warranted echocardiographic evaluation to exclude endocarditis, independent of the elevated natriuretic peptide that ultimately prompted imaging in this case.

## 5. Management and Clinical Course

### 5.1. Patient Course and Outcome

The patient completed a 6‐week course of intravenous vancomycin. Tigecycline was administered for 14 days prior to lead extraction and for a further 5 days following extraction. The antimicrobial regimen represented broad empirical coverage in culture‐negative disease; tigecycline was used in this patient due to antibiotic allergies. This is stated explicitly because tigecycline is not a standard first‐line agent for endovascular infection, owing to the low serum concentrations achieved, and its use, therefore, warrants justification. Repeat blood cultures were negative. The infected system was removed by transvenous (percutaneous) lead extraction at the specialist centre. Following extraction, the patient was considered unsuitable for open cardiac surgery. Reimplantation of a permanent pacemaker was deferred unless symptoms recurred and could not otherwise be controlled, and no routine cardiology follow‐up was arranged.

### 5.2. Device Extraction and Antibiotic‐Duration Principles

In confirmed systemic CIED infection (e.g. CIED‐related IE), complete system extraction is recommended and should be performed in centres with appropriate procedural expertise [1, 4]. In the present case, NT‐proBNP was significantly increased (3382 ng/L). This is known to be a marker of worse prognosis and increased mortality in patients with IE [7, 8], and this contributed towards the decision for timely extraction of the infected system. Postextraction antibiotic duration is individualised to the organism (if known), the presence or absence of valvular involvement, clinical complications (e.g. septic emboli) and the extent of intracardiac infection; longer treatment courses are generally expected when intracardiac vegetations or complicated infection is present [1, 4].

### 5.3. Large Right‐Sided Vegetations and Extraction Strategy

The patient′s vegetation size (~2.5 cm) is clinically relevant. Larger right‐sided lead vegetations may increase concern for pulmonary embolisation during extraction. Prospective imaging studies of lead‐related IE demonstrate a high prevalence of (often clinically silent) pulmonary emboli in patients with lead vegetations, and observational data suggest that larger vegetations (e.g. ≥ 20 mm) may increase embolic risk [9].

When large vegetations are present, some centres consider adjunctive approaches such as percutaneous aspiration/debulking prior to or during transvenous lead extraction, aiming to reduce embolic burden and facilitate safer extraction; multicentre series describe feasibility and procedural success, although evidence remains largely observational and patient selection is individualised [10].

### 5.4. Diagnostic Synthesis for Echocardiography Practice

This case (summarised in Table 1) illustrates a common diagnostic pattern in systemic CIED infection: Apparent improvement of an alternative diagnosis (e.g. pneumonia) should not lower suspicion when fever and inflammation persist. In CIED recipients, persistent systemic symptoms warrant careful evaluation for right‐sided CIED‐related IE, including dedicated echocardiographic assessment of intracardiac leads [1, 4].

**Table 1:** Clinical timeline of the case, from presentation to outcome.

Stage/event	Detail
Presentation	Adult woman with a permanent pacemaker; fever, pleuritic chest pain, productive (yellow) sputum. Background: COPD, Type 2 diabetes, hypertension, psychiatric comorbidity.
Initial diagnosis	Right‐sided community‐acquired pneumonia; treated with clinical improvement.
In‐hospital complication	*Clostridioides difficile*–positive diarrhoea; resolved with therapy.
Persistent signs	Ongoing pyrexia and persistently raised inflammatory markers despite respiratory improvement.
Trigger for echo	Shortness of breath + pleural effusion on CXR → NT‐proBNP markedly elevated (3382 ng/L) → inpatient TTE.
Microbiology	Multiple blood cultures negative (antecedent and ongoing antimicrobial therapy).
TTE (late Dec 2025)	Mobile, irregular lead‐adherent right atrial mass (≈2.3 × 1.0 cm) prolapsing across the tricuspid valve in diastole; mild TR; LVEF 58%; raised LV filling pressures.
TOE	Lead‐adherent mass (≈2.5 × 1.0 cm), irregular and independently mobile; mild–moderate eccentric TR; interpreted as vegetation.
Diagnosis	Possible CIED‐related infective endocarditis (2023 Duke–ISCVID: 1 major + 2 minor criteria).
Management	IV vancomycin for 6 weeks; tigecycline for 14 days before and 5 days after extraction; transvenous lead extraction at a specialist centre.
Outcome	Repeat blood cultures negative; not a candidate for open cardiac surgery; pacemaker reimplantation deferred; no routine cardiology follow‐up.

From an echocardiography standpoint, the educational value here lies in (1) recognising the appearance and motion of a mobile lead‐adherent right atrial mass prolapsing through the tricuspid valve, (2) being aware of the most common etiologies of CIED lead‐adherent masses and (3) being aware of the importance of the overall clinical context and always taking this into consideration when reporting abnormal findings [1, 5, 6].

### 5.5. BCNE Evaluation and the Role of Advanced Imaging

In BCNE, modern diagnostic recommendations support a structured work‐up adapted to epidemiology and exposures, commonly incorporating targeted serology and molecular diagnostics (e.g. PCR/sequencing) and emphasising tissue or device material testing when available [2, 3]. The 2023 Duke–ISCVID criteria and the AHA BCNE statement both reflect the growing role of molecular microbiology and advanced imaging in strengthening diagnostic classification and guiding management in culture‐negative cases [2, 3].

The absence of microbiological testing of the explanted material is addressed in the Limitations section below.

Multimodality imaging can be helpful when echocardiographic findings are equivocal or when diagnostic certainty remains limited. ESC guidelines include FDG PET/CT (and/or labelled white blood cell SPECT/CT) as adjuncts in suspected CIED‐related IE and in complex presentations, acknowledging that imaging should be interpreted alongside clinical and microbiologic data [1].

## 6. Limitations

Microbiological testing (PCR and/or culture) of the explanted lead/vegetation was not performed. This represents a limitation of the present case, as molecular and tissue/device testing of explanted material is the recommended diagnostic pathway in culture‐negative disease, and its absence precludes pathogen identification and a definite, pathologically confirmed classification of IE. Additionally, it could not be confirmed whether the pacemaker pocket was examined for local infection during this admission, which presents a further limitation of the present case.

## 7. Learning Points

• Persistent fever in patients with CIEDs should prompt evaluation for systemic device infection even when an apparent extracardiac infection improves.
• Echocardiography (both TTE and TOE) is central for detecting and characterising lead‐associated intracardiac masses and assessing potential damage to cardiac structures.
• Lead‐associated echodensities are not always synonymous with infection; diagnosis requires integration of imaging findings with systemic inflammatory features and microbiologic data.
• BCNE requires a structured diagnostic approach and early consideration of antimicrobial toxicity, with monitoring for treatment‐related toxicity during prolonged antimicrobial therapy.

## Funding

No funding was received for this manuscript.

## Ethics Statement

Ethical approval was granted for this case report.

## Consent

Written informed consent was obtained from the patient for publication of this case report and accompanying images.

## Conflicts of Interest

The authors declare no conflicts of interest.

## Data Availability

Data sharing is not applicable to this article, as no datasets were generated or analysed during the current study.
